# Dronedarone hydrochloride (DH) induces pancreatic cancer cell death by triggering mtDNA-mediated pyroptosis

**DOI:** 10.1038/s41419-024-07102-w

**Published:** 2024-10-02

**Authors:** Ming-Qiao Li, Yu-Qi He, Meng-Ni Zhang, Wan Tang, Ya Tan, Yue Cheng, Mei Yang, Nan Zhao, Ling Li, Si-Rui Yu, Ruo-Lan Li, Qiong Pan, Ming-Yue Wu, Jin Chai

**Affiliations:** 1 Department of Gastroenterology, the First Affiliated Hospital (Southwest Hospital), Third Military Medical University (Army Medical University), Chongqing, 400038 China; 2 Institute of Digestive Diseases of PLA, Third Military Medical University (Army Medical University), Chongqing, 400038 China; 3 Cholestatic Liver Diseases Center, the First Affiliated Hospital (Southwest Hospital), Third Military Medical University (Army Medical University), Chongqing, 400038 China; 4 Metabolic Dysfunction-Associated Fatty Liver Disease (MASLD), the First Affiliated Hospital (Southwest Hospital), Third Military Medical University (Army Medical University), 400038 Chongqing, China

**Keywords:** Pharmacology, Gastrointestinal cancer, Cell death

## Abstract

Pancreatic cancer is one of the leading causes of cancer-associated mortality, with a poor treatment approach. Previous study has shown that inducing pyroptosis in pancreatic ductal adenocarcinoma (PDAC) slows the growth of PDACs, implying that pyroptosis inducers are potentially effective for PDAC therapy. Here, we found that Dronedarone hydrochloride (DH), an antiarrhythmic drug, induces pyroptosis in pancreatic cancer cells and inhibits PDAC development in mice. In PANC-1 cells, DH caused cell death in a dosage- and time-dependent manner, with only pyroptosis inhibitors and GSDMD silencing rescuing the cell death, indicating that DH triggered GSDMD-dependent pyroptosis. Further work revealed that DH increased mitochondrial stresses and caused mitochondrial DNA (mtDNA) leakage, activating the cytosolic STING-cGAS and pyroptosis pathways. Finally, we assessed the anti-cancer effects of DH in a pancreatic cancer mouse model and found that DH treatment suppressed pancreatic tumor development in vivo. Collectively, our investigation demonstrates that DH triggers pyroptosis in PDAC and proposes its potential effects on anti-PDAC growth.

## Introduction

Pancreatic cancer is one of the leading causes of cancer mortality, with a 5-year survival rate of 8% [1]. There is an urgent need for the study of advanced therapeutic strategies and the identification of novel, effective chemotherapy drugs for pancreatic cancer treatment. Previous study has indicated that the induction of pyroptosis suppressed PDAC proliferation and inhibited pancreatic tumor growth in vivo, suggesting that triggering pyroptosis is a potent approach for pancreatic cancer therapy [2].

Pyroptosis is a type of programmed cell death induced by formation of gasdermins-mediated membrane pores [3]. Initially regarded as a protective process for the host to prevent immune cells from pathogen insult, emerging evidence has proven that pyroptosis also occurs in tissue or cancer cells in response to a range of stimuli [4]. Triggering pyroptosis in cancer cells has emerged as a promising strategy for cancer treatment due to its ability to expedite cancer cell death [5]. Interestingly, fluorouracil (5-FU), a chemotherapy agent for PDAC, has been proved to activate pyroptosis [6], implying that novel pyroptosis inducers may be a perspective agent for PDAC treatment.

Dronedarone hydrochloride (DH), a noniodinated benzofuran analog of amiodarone, is an FDA-approved antiarrhythmic drug used to treat atrial fibrillation [7]. Mechanistic studies have demonstrated that DH inhibits multiple cardiac ion channels, decreasing dispersion of repolarization between ventricular epicardial, endocardial M cells and Purkinje fibres [8]. Furthermore, a previous study noted that DH also had cytotoxic effects in cancer cells [9], revealing its anti-cancer properties. However, the mechanism of DH-induced cytotoxicity and whether it acts in PDAC remain elusive.

In this study, we have found that the antiarrhythmic drug, DH, can induce rapid and severe cell damage in PDAC by triggering pyrotosis. In PANC-1, a pancreatic cancer cell line, DH induced the release of mitochondria DNA (mtDNA) and led to inflammasome-mediated gasdermin D (GSDMD) activation, ultimately activating pyroptosis and inhibiting pancreatic tumor growth in vivo. These findings have uncovered a novel mechanism by which DH exhibits cytotoxicity against pancreatic cancer cells and have suggested the possibility of using DH in the treatment of pancreatic cancer.

## Results

### Dronedarone hydrochloride (DH) induces cell death in pancreatic cancer cells

**Fig. 1:**
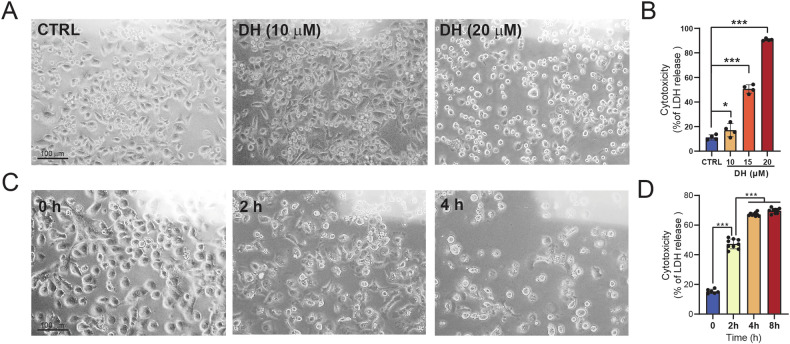
DH induced pancreatic cancer cell death. **A** Morphology observation of PANC-1 after treatment of DH at different concentrations. Scale bar, 100 μm. **B** Released LDH activity in PANC-1 cells after treatment of DH at different concentrations. *n* = 4. **C** Morphology observation of PANC-1 after treatment of DH (20 μM) at indicated time point. Scale bar, 100 μm. **D** Released LDH activity in PANC-1 cells after treatment of DH (20 μM) at indicated time point. *n* = 8. Data was shown as mean ± SD. Mutiple *t*-test are followed by Bonferoni correction was used. *p < 0.05; ***p < 0.001.

Although DH is utilized as an antiarrhythmic drug, fewer studies have focused on its cytotoxic effects on pancreatic cancer cells. Our previous study demonstrated that DH induces cell death at indicated concentrations (data not shown). We hypothesize that DH may also cause pancreatic cancer cell death in a similar manner. As expected, DH exhibited significant toxicity on the pancreatic cell lines after 12 h (Fig. 1A, B, Supplementary Fig. 1A). As shown in Fig. 1A, cell morphologies were noticeably altered, and cellular lactate dehydrogenase (LDH) was dramatically released from cell (Fig. 1B). Moreover, we found that 20 μM DH treatment began to induce cytotoxicity after 2 h and reached its maximum cytotoxic level after 8 h (Fig. 1C, D). Overall, DH was shown to induce cell toxicity in PANC-1 cells in a dose- and time-dependent manner.

### DH induces pyroptosis in pancreatic cancer cell lines

**Fig. 2:**
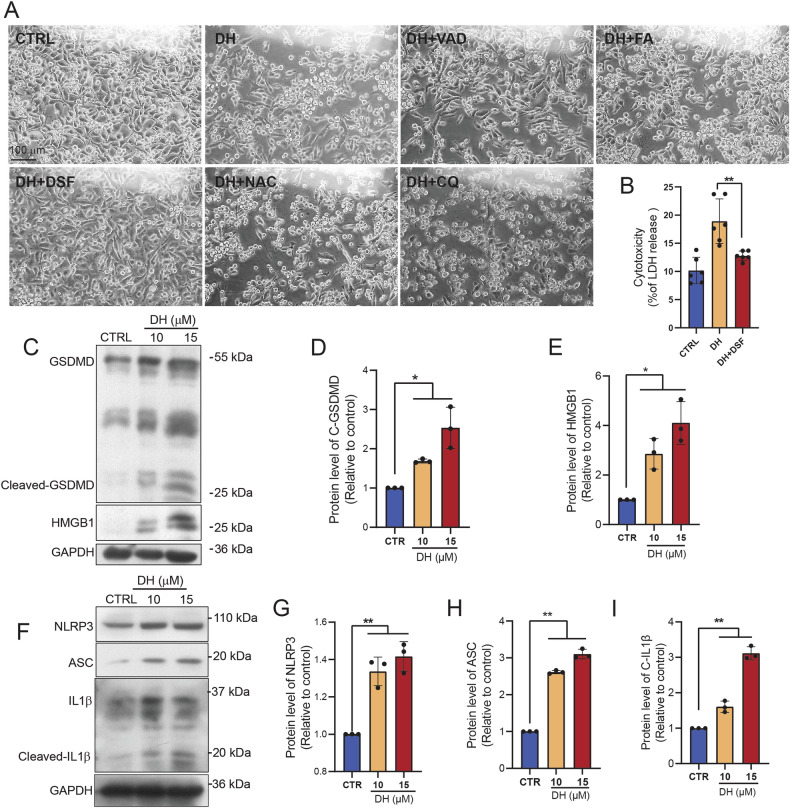
DH triggered inflammasome-related pyroptosis. **A** Morphology observation of PANC-1 after treatment of vehicle, DH (15 μM) or DH combined with different inhibitors. Z-VAD-FMK (VAD), pan-caspase inhibitors; Z-FA-FMK (FA), cathepsin B/L inhibitor; disulfiram (DSF), pyroptosis inhibitor; N-acetyl-Lcysteine (NAC), ROS inhibitor; chloroquine (CQ), autophagy inhibitor; Scale bar, 100 μm. **B** Released LDH activity in PANC-1 cells after treatment of DH or DH combined with DSF. *n* = 6. **C** Representative Western Blot images of pyroptosis-related proteins expression in PANC-1 cells after DH treatment. **D**, **E** Quantification of Cleaved-GSDMD, HMGB1 protein expressions in Fig. 2C. *n* = 3. **F** Representative Western Blot images of inflammasome-related proteins expression in PANC-1 cells after DH treatment. **G**–**I** Quantification of NLRP3, ASC and cleaved-IL1β protein expressions in Fig. 2F. *n* = 3. Data was shown as mean ± SD. Mutiple *t*-test are followed by Bonferoni correction was used. *p < 0.05; **p < 0.01.

To investigate the types of cell death triggered by DH, we pretreated PANC-1 cells with inhibitors targeting different cell death pathways. We found that only pyroptosis inhibitor, disulfiram (DSF), obviously block DH-induced cell death, indicating that DH triggered pyroptosis-dependent cell death (Fig. 2A, B; Supplementary Fig. 3F). Pyroptosis is usually induced by GSDMD cleavage, which drives plasma membrane pore formation and accelerates cell lytic [3]. NLRP3/ASC inflammasome activation is one of the major mechanisms to trigger pyroptosis by inducing caspase-1-mediated GSDMD cleavage [10]. As expected, we found that DH induced higher expression of cleaved-GSDMD and HMGB1 compared to the control in PANC-1 or Capan-2 cells (Fig. 2C–E, Supplementary Fig. 1B). Western Blot analysis revealed that DH induced a time-dependent increase of cleaved GSDMD and HMGB1 (Supplementary Fig. 1C–E). The pan-caspase inhibitors, Z-VAD-FMK, didn’t reverse the DH-induced cell death and GSDMD activation, indicating a non-canonical pyroptosis pathway has been triggered (Supplementary Fig. 3L–N). Additionally, DH promoted the translocation of nuclear HMGB1 to cytosol in PANC1 cell lines (Supplementary Fig. 1F). According to our observation, we found DH also induced pyroptosis in non-PDAC cells, such as RAW 264.7 cells (Supplementary Fig. 1G–I). Furthermore, the expression of inflammasome-related proteins, such as NLRP3, ACS and cleaved IL-1β, was also promoted by DH in PANC-1 cells (Fig. 2F–I). Taken together, DH treatment activated an inflammasome-related pyroptotsis pathway in PDAC cells.

### DH-induced pyroptosis was dependent on cleavage of GSDM family

**Fig. 3:**
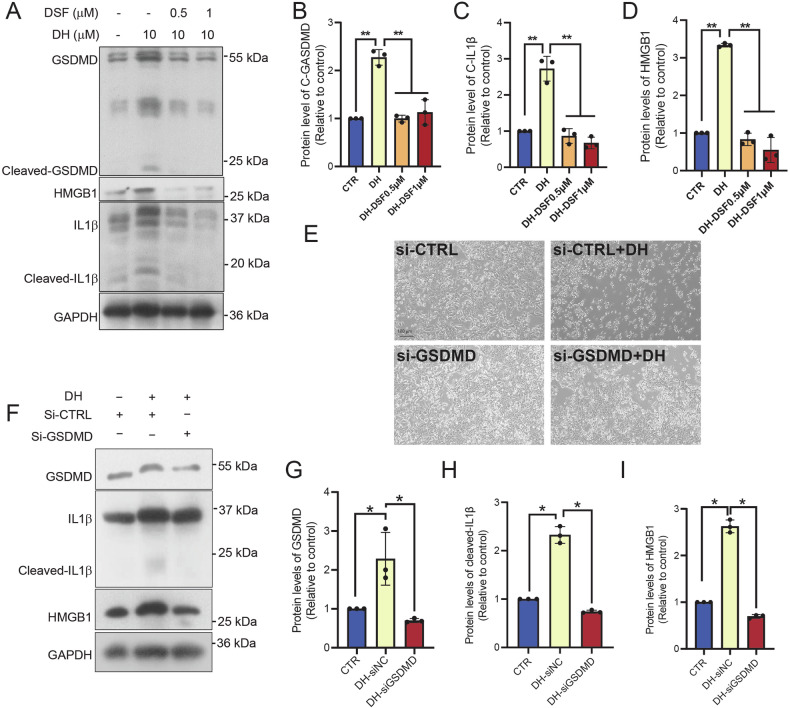
Inhibition of GSDMD blocked DH-induced cell death. **A** Representative images of Western Blot images of pyroptosis-related proteins in PANC-1 cells after DH or DH combined DSF treatment. **B**–**D** Quantification of cleaved-GSDMD, HMGB1, and cleaved-IL1β protein expressions in Fig. 3A. *n* = 3. **E** Morphology observation of PANC-1 after treatment of vehicle or DH. GSDMD was knocked down or not in cells before DH treatment. Scale bar, 100 μm. **F** Representative images of Western Blot images of pyroptosis-related proteins in cells with different treatment. **G**–**I** Quantification of cleaved-GSDMD, HMGB1, and cleaved-IL1β protein expressions in Fig. 3F. *n* = 3. Data was shown as mean ± SD. Mutiple *t*-test are followed by Bonferoni correction was used. *p < 0.05; **p < 0.01.

The gasdermin (GSDM) family of proteins plays major role in mediating pyroptosis. We identified GSDMB, GSDMD, and GSDME as the top three genes expressed in pancreatic cancer (Supplementary Fig. 3B). To investigate whether they are the pyroptosis executioner, we blocked the pyroptosis signaling by adding disulfiram (DSF) firstly, a GSDMD inhibitor. We found that DSF totally block DH-induced cleaved-GSDMD, HMGB1, and cleaved-IL1β expression in PANC-1 cells (Fig. 3A–D). Furthermore, we genetically silenced GSDMD expression using siRNA. Similarly, knocking down GSDMD in PANC-1 cells obviously inhibited DH-induced cell death when comparing cell morphology (Fig. 3E). In addition, lower expression of cleaved-IL-1β and HMGB1 were observed in GSDMD knockdown cells with different siRNA oligos after DH treatment (Fig. 3F–I; Supplementary Fig. 3G–K), indicating DH induced a GSDMD-dependent pyroptosis. Given that GSDMB and GSDME are also highly expressed in pancreatic cancer (Supplementary Fig. 3B). To this end, we silenced GSDMB and GSDME expression. Interestingly, our results found that both GSDMB and GSMDE, similar to GSDMD, are involved in DH-induced pancreatic cell death. These results demonstrate that DH-induced cell death is dependent on GSDM family, but not a specific one (Supplementary Fig. 3C–E).

### DH disturbs mitochondrial homeostasis

**Fig. 4:**
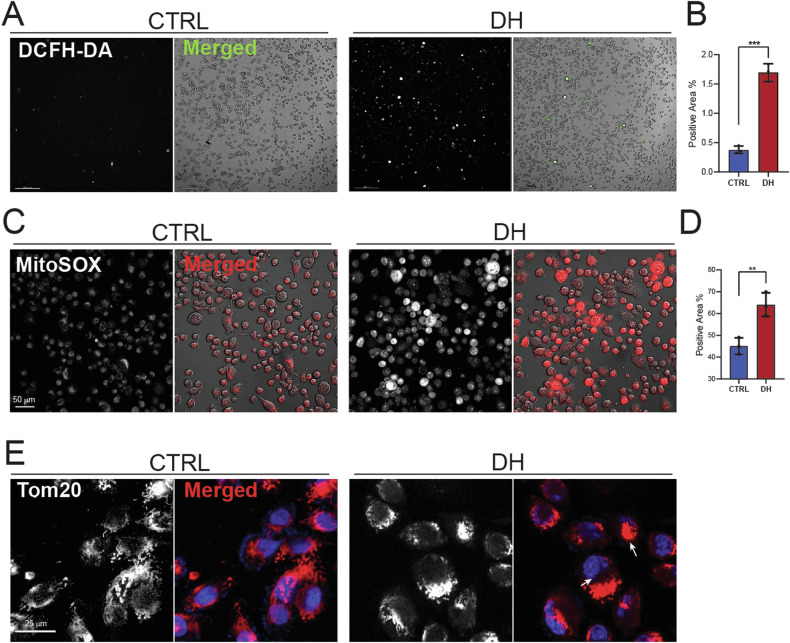
DH disturbed mitochondria homeostasis. **A** Representative images of DCFH-FA staining result in PANC-1 cells with or without DH treatment. White or Green, DCFH positive stained area. Scale bar, 200 μm. **B** Quantification of DCFH-FA positive staining area in images in Fig. 4A. *n* = 3. **C** Representative images of MitoSox staining results in PANC-1 cells with or without DH treatment. Scale bar, 50 μm. Red, MitoSox positive stained area. **D** Quantification of MitoSox positive staining area in images in Fig. 4C. *n* = 3. **E** Representative immunofluorescent images of Tom20 staining results in PANC-1 cells with or without DH treatment. Scale bar, 25 μm. Red, Tom20; Blue, DAPI. Data was shown as mean ± SD. Unpaired *t*-test was used. **p < 0.01; ***p < 0.001.

DH has been revealed to act as an uncoupler and inhibitor of the respiratory chain in mitochondria, leading to an accumulation of reactive oxygen species (ROS) and resulting in cell death [11]. To investigate whether DH induces ROS accumulation and mitochondria dysfunction prior to displaying severe cytotoxicity, we detected cellular ROS, mt-ROS, and mitochondria morphologies using different probes. The intracellular ROS signal was obviously higher after DH treatment, as indicated by the results of DCFH assay (Fig. 4A, B). Mitochondrial ROS generation was assessed using MitoSOX, an mt-ROS probe. The results showed that PANC1 cells, particularly the dying cells, displayed a robust mt-ROS signal in response to DH (Fig. 4C, D). In addition, our time-lapse imaging results revealed that mitochondrial ROS are generated before cell death occurs (Supplementary Fig. 2A and Supplementary video 1). To assess mitochondrial morphology alterations, we used TOM20 to stain mitochondria in PANC1 cells. The results showed that DH stimulated mitochondrial accumulation, indicating a disruption in mitochondrial homeostasis (Fig. 4E). Collectively, DH induced a robust mt-ROS generation and disrupted mitochondrial homeostasis before triggering cell death.

### DH-induced mitochondrial DNA (mtDNA)-mediated pyroptosis

**Fig. 5:**
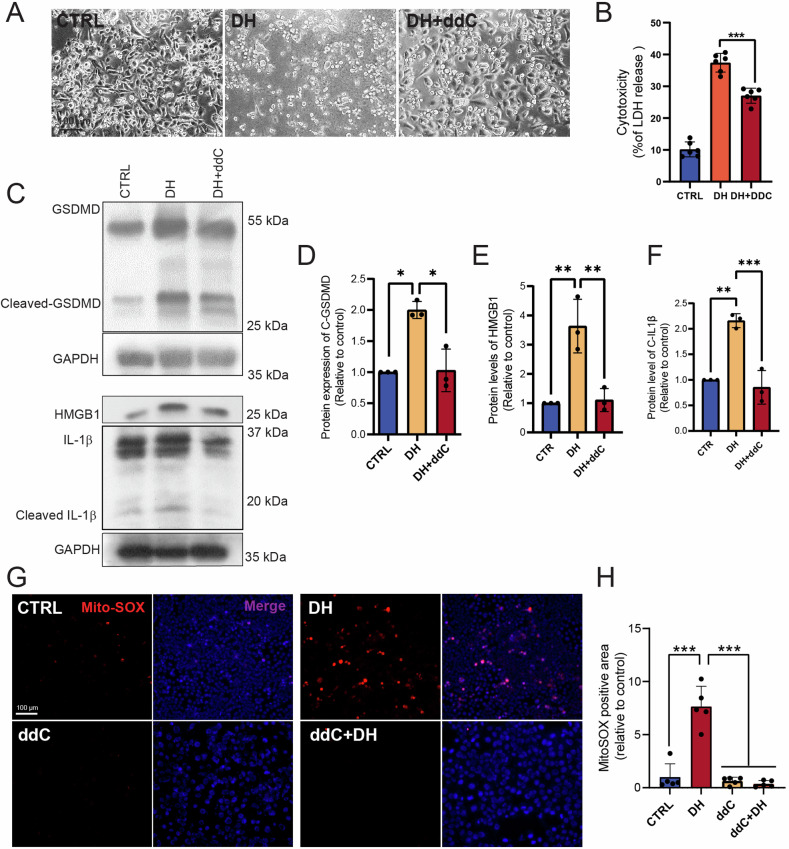
DH-induced mitochondrial DNA (mtDNA)-mediated pyroptosis. **A** Morphology observation of PANC-1 after treatment of vehicle, DH or DH combined ddC. Scale bar, 100 μm. **B** Released LDH activity in PANC-1 cells after treatment of DH or DH combined with ddC. *n* = 6. **C** Representative images of Western Blot images of pyroptosis-related proteins in PANC-1 cells after different treatment. **D**–**F** Quantification of cleaved-GSDMD, HMGB1, and cleaved-IL1β protein expressions in Fig. 5C. *n* = 3. **G** Representative images of MitoSox staining results in PANC-1 cells after different treatments. Scale bar, 100 μm. Red, MitoSOX; Blue, DAPI. **H** Quantification of MitoSOX positive staining area in images in figure (**G**). *n* = 5. Data was shown as mean ± SD. Mutiple *t*-test are followed by Bonferoni correction was used. *p < 0.05; **p < 0.01; ***p < 0.001.

Mitochondrial DNA (mtDNA) released from damaged mitochondria stimulates inflammasome activation, resulting in pyroptosis. To investigate whether DH-induced mitochondrial dysfunction can cause mtDNA release and inflammasome-pyroptosis signaling, we pre-treated PANC1 cells with 2’,3’-dideoxycytidine (ddC) to delete cellular mtDNA [12]. The immunofluorescence images revealed that cytosolic DNA was significantly reduced in ddC-treated PANC1 cells (Supplementary Fig. 2B, C). Pretreatment with ddC markedly protected cells from DH-induced cell death (Fig. 5A, B; Supplementary Fig. 3F). Furthermore, ddC treatment effectively suppressed DH-induced protein expression of pyroptosis-related proteins such as cleaved-GSDMD, HMGB1, cleaved-IL-1β (Fig. 5C–F). To investigate whether mtDNA release was prior to DH-induced ROS, we monitored ROS levels following ddC treatment. The results showed that DH-induced ROS can be completely inhibited by ddC pretreatment (Fig. 5G, H), demonstrating that ROS stimulation is a downstream effect of mtDNA release that not directly related to GSDMD activation. These results suggest that DH treatment disrupted mitochondrial homeostasis, possibly leading to mtDNA-related cellular stress and pyroptosis in PANC1 cells. Additionally, the results demonstrated that mtDNA is the trigger of DH-related pyroptosis.

### DH triggers mtDNA release and induces cGAS-STING activation

**Fig. 6:**
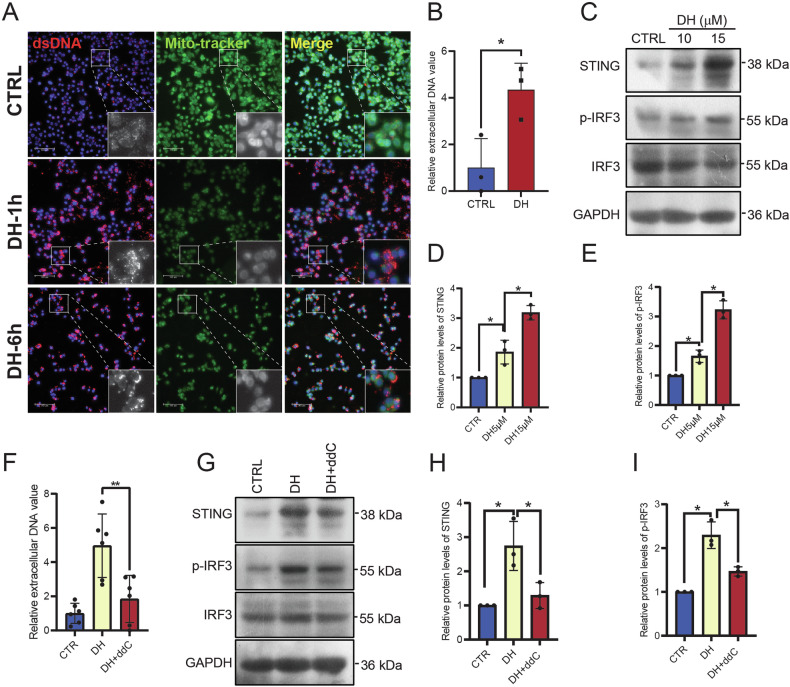
DH induced mtDNA release and triggered cGAS-STING pathway. **A** Representative images of PANC1 cells after stained with dsDNA and MitoTracker. Green, MitoTracker; Red, dsDNA. Scale bar, 100 μm. **B** Determination of extracellular DNA value in the culture medium of PANC-1 cells after DH treatment. *n* = 3. **C** Representative images of Western Blot images of cGAS-STING pathway-related proteins in PANC-1 cells after different treatment. **D**, **E** Quantification of STING, p-IRF3 protein expressions in Fig. 6B. *n* = 3. **F** Determination of extracellular DNA value in the culture medium of PANC-1 cells after DH or DH combined ddC treatment. *n* = 6. **G** Representative images of Western Blot images of cGAS-STING pathway-related proteins in PANC-1 cells after DH or DH combined ddC treatment. **H**, **I** Quantification of STING, p-IRF3 protein expressions in Fig. 6F. *n* = 3. Data was shown as mean ± SD. Mutiple *t*-test are followed by Bonferoni correction was used. *p < 0.05.

Cytosolic mitochondrial DNA (mtDNA), also known as cytosolic double-stranded DNA (dsDNA), is a potent DAMP that can trigger innate immune responses via activating cGAS-STING pathway [13]. We hypothesized that DH triggers mtDNA leakage, inducing pyroptosis and increasing cGAS-STING signaling. To validate our hypothesis, we co-stained dsDNA and MitoTracker to localize mtDNA following DH treatment. We found cytosolic DNA was dispersed in control cells, but formed DNA aggregation after one hour of DH treatment (Fig. 6A). Some of these DNA aggregates colocalized with MitoTracker. Additionally, we noted a reduction in cytosolic DNA, indicating an increased release of DNA (Fig. 6A). Subsequently, we detected the extracellular DNA content by staining DNA with a nuclear dye. The result showed that DH treatment induced more DNA leakage from cells (Fig. 6B). STING and p-IRF3 are two important regulating proteins in cGAS-STING signaling. The results revealed that DH treatment increased the expression of STING and p-IRF3 (Fig. 6C–E), indicating that more cytosolic DNA was released. However, the inhibition of mtDNA production by adding ddC blocked extracellular DNA release (Fig. 6F) and cGAS-STING signal (Fig. 6G–I). To figure out whether mtDNA released into cytosol activates cGAS, we employed cGAS-chromatin immunoprecipitation followed by qPCR analysis of cGAS-bound mtDNA. The results showed a significant increase in the co-precipitation of mtDNA with cGAS following DH treatment (Supplementary Fig. 3A). These results demonstrated that DH-induced-mtDNA leakage is the trigger of GSDMD-dependent pyroptosis.

### DH inhibits pancreatic tumor growth in vivo

**Fig. 7:**
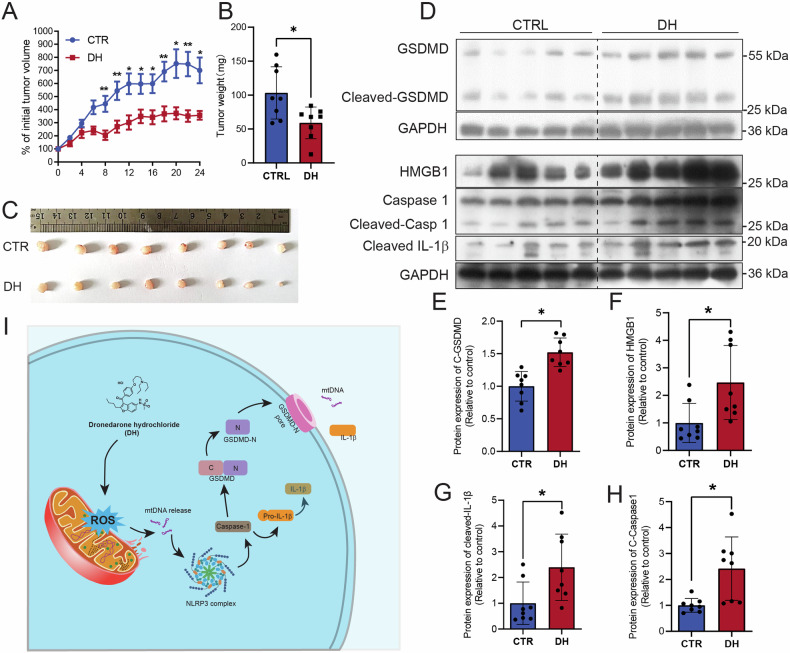
DH treatment inhibit pancreatic tumor growth in vivo. **A** Relative tumor growth rate after DH treatment. *n* = 9,10. **B** Comparison of tumor weight after DH treatment. *n* = 8. **C** Representative images of tumors from mice with DH treatment or not. *n* = 8. **D** Representative images of Western Blot images of pyroptosis-related proteins in tumors from mice with or without DH treatment. **E**–**H** Quantification of cleaved-GSDMD, HMGB1, cleaved-caspase 1, and cleaved-IL1β protein expressions in (**D**). **I** A diagram illustrating working machinery of DH on stimulation of pyroptosis. Data was shown as mean ± SD. Mutiple *t*-test are followed by Bonferoni correction was used. *p < 0.05; **p < 0.01; ***p < 0.001.

To investigate whether DH-induced pyroptosis contributes to the inhibition of PADC growth, we established a mouse xenograft model using pancreatic cancer cells. The results showed that DH treatment inhibited tumor growth in mice (Fig. 7A–C). DH treatment enhanced the expression of cleaved-GSDMD, HMGB1, cleaved-Caspase1, cleaved IL-1β (Figure D–H). As previous studies have reported adverse effects of DH on liver function in previous study [14], we further determined the organ weight and liver function in our mice model. The results showed that DH treatment had no effect on liver, kidney, spleen weight index and kidney function (Supplementary Fig. 4B–D, G), but did result in lower body weight (Supplementary Fig. 4A) and somewhat increase liver function (Supplementary Fig. 4E, F). The HE staining results also showed that DH treatment aggravated the infiltration of inflammatory cells, which is consistent to liver function test (Supplementary Fig. 4H).

## Discussion

DH is a clinical drug used to treat atrial fibrillation that has been thought to have fewer side effects. In our study, we unraveled a novel biological function of DH that causes pancreatic cancer cell death by inducing mtDNA-mediated pyroptosis. We found that DH induces tremendous pancreatic cancer cell death within a short time at a dose of 15 μM or above. Inhibition of GSDMD cleavage by adding specific inhibitor blocked DH-induced cell death, indicating DH potentially induced pyroptosis in pancreatic cancer cells. Further evidence proved that DH stimulated mitochondrial dysfunction, which led to mitochondrial ROS production and mtDNA release. Depletion of mtDNA with ddT inhibit DH-stimulated pyroptosis, implying that the released mtDNA was a major activator of inflammasome-associated pyroptosis following DH treatment. Accumulating studies have revealed the potential roles of pyroptosis in regulating cancer cell proliferation [4]. To evaluate whether DH affects pancreatic cancer cell growth in vivo, we examined the growth of PANC-1 xenografted tumors in nude mice treated with DH. The results showed that DH treatment inhibited cancer cell growth to a certain extent. Collectively, this is the first study to unravel the roles of DH in initiating pyroptosis in pancreatic cancer cells by disrupting mitochondrial homeostasis and generating mtDNA release.

A previous study has found that DH may have anti-breast cancer benefits since it antagonizes thyroid hormone receptors (THRs) [9]. However, the study concluded that the DH-induced cytotoxicity was independent of THR antagonism, as depletion of THR didn’t reverse the effects, indicating involvement of another pathway. In our study, we proposed a novel anti-cancer mechanism of DH, in which DH inhibited tumor growth by triggering pyroptosis in cancer cells. Inhibition of GSDMD cleavage and depletion of mitochondria mtDNA both prevented DH-indcued cell death, implying an mtDNA-mediated pyroptosis pathway. Our findings expanded our understanding of the mechanism by which DH induces cancer cell death. To determine whether the effects of DH-induced cell death varies across different cell types, we treated DH on both normal and cancer cell lines. However, the results showed that DH-induced cell death is not specific to any particular cell type (Data was not shown).

Recently, the concept of cleavage is the only trigger for GSDMD was been challenged by a recent study [15]. Reversible palmitoylation of GSDMD has been identified as a crucial checkpoint for pore formation. Moreover, modifications such as O-GlcNAc and succination have been implicated in directing GSDMD functions [16, 17]. These findings suggest that protein modification of GSDMD play a significant role in its biological activity. Future studies should investigate whether DH influences translational modification. Moreover, DH treatment also led to increased expression levels of NLRP3, ASC and STING. We hypothesize that these enhancements may result from a reduced degradation pathway following DH treatment (Data was not shown).

GSDMD is hypothesized to be cleaved by different caspases, including caspase 1, 4 or 11 [3]. However, we found that DH-induced GSDMD activation cannot be rescued by adding z-VAD, a well-known pan-caspase inhibitor. The findings suggested a noncanonical process involved in, if not another undiscovered cellular function of z-VAD. In fact, a unique pyroptosis regulatory mechanism that independent of caspases-dependent cleave has just been revealed recently [15]. Palmitoylation of GSDMD induces pyroptosis by accelerating liposome leakage and forms pores. Our findings provoke in-depth mechanism consideration for DH-induced pyroptosis.

Identification of pyroptosis inducers has recently been suggested as a promising strategy for inhibiting cancer progression [5]. In fact, several small molecules, such as Chalcone analogue 8 [18], thiopyran derivative L61H10 [19], diosin [20], and galangin [21] have been designed or identified to execute pyroptosis in cancer cells. DH, a novel pyroptosis inducer, has the benefit of being an anti-cancer drug, since it has already been employed in clinical practice. However, the adverse effects of DH are the major impediment to its future adoption. Our study showed that DH treatment reduced mice’s body weight and liver function. More research is needed to evaluate whether DH is appropriate for pancreatic cancer treatment in a clinical trial.

## Materials and methods

### Reagents and antibodies

Dronedarone hydrochloride (DH) (D808235) was obtained in Macklin Inc (Shanghai, China). Disulfram (DSF) (HY-B0240), Z-FA-FMK (HY-P01094) and Chloroquine (CQ) (HY-17589A) were purchased from MedChemExpress (Shanghai, China). 2’,7’-Dichlorofluorescein diacetate (DCFH-DA) (4091-99-0), 2’,3’-dideoxycytidine (ddC) (7481-89-2) was obtained from Aladdin Industrial Corporation (Shanghai, China). The transfection kit was purchased from Biomed (Beijing, China). Lactate dehydrogenase (LDH) kit (C006), Z-VAD-FMK (C1202), NAC (S0077) and DAPI (C1005) were obtained from Beyotime Biotechnology (Shanghai, China). MitoSOX (M36009) and MitoTracker (M7514) were purchased from ThermoFisher Scientific (MA, USA). TB Green (RR802A) was obtained from Takara Biomedical Technology (Beijing, China). Protein A/G magnetic beads (HY-K0202) were purchased from MedChemExpress (USA).

Anti-Tom20 antibody (382451) was obtained from ZEN-BIOSCIENCE (Chengdu, China). HRP goat anti-rabbit IgG antibody (AB205718) was purchased from Abcam (MA, USA). Goat anti-rabbit IgG (Alexa Fluor 555 Conjugate) (4413S), anti-p-IRF3 antibody (29047t), anti-IRF3 antibody (11904t), anti-STING antibody (13647t), anti-NLRP3 antibody (15101 s), anti-ASC antibody (67824t), and anti-IL-1β antibody were purchased from Cell Signaling Technology (MA, USA). Anti-GSDMD antibody (WH322930), anti-HMGB1 antibody (A2553) and anti-GAPDH antibody (A19056) were obtained from ABclonal Technology (Wuhan, China). Anti-DNA antibody (61014) was purchased from Progen Biotechnik (Germany). Anti-cGAS antibody (26416-1-AP) was obtained from Proteintech (China).

### Animals

Four-week-old male BALB/cAJcl-nu mice were purchased from HuaFuKang (HFK Bioscience, China) and housed under controlled temperature (25 °C) on 12 h light-dark cycle, with *ad libitum* access to food and water. All animal procedures were performed according to the guidelines of the Animal Care and Use Committees at the Medical Research Center. The study protocol was reviewed and approved by the institutional Animal Care and Use Committee of the Southwest Hospital, Chongqing, China. The xenograft tumor model was generated by subcutaneous injection of PANC-1 cells (3 × 10^6^ cells per mice) in the right flank of each mouse. Mice received an intraperitoneal injection (i.p.) injection of either vehicle or 50 mg/kg DH (DH dissolved in 90% olive oil) every two days. Body weight and size of tumors were measured every two days. The tumor volume was calculated by the formula: tumor volume V (mm^3^) = 1/2 x length (mm) x width (mm)^2^. Mice were sacrificed after 24 days. The weight of tumor, liver, kidney, and spleen were measured and recorded. Serum was collected for determination of liver and kidney function in mice.

### Cell culture

Human pancreatic adenocarcinoma PANC-1, mouse Raw 264.7 and Capan-2 cell lines were purchased from Hunan Fenghui Biotechnology Co., Ltd. (Hunan, China). Cells were maintained in Dulbecco’s modified Eagle’s medium (DMEM, Gibco) supplemented with 10% fetal bovine serum (Gibco) and 50 μg/ml penicillin and streptomycin (Gibco).

### Morhological assessment of cells

PANC-1, Capan-2, RAW 264.7 cells were seeded at a density of 1 × 10^5^ per well in a 24-well plate and incubated at 37 °C in an incubator supplied with 5% CO_2_. After 12 h, cells were treated with DH or other inhibitors, and further incubated for indicated time points. For ddC combined DH treatment, cells were pretreated with ddC for 36 h before adding DH. The cell morphology was observed under a phase-contrast inverted microscope.

### Transfection of siRNA

PANC-1 cells were plated into a 6-well dish and transfected with siRNAs in serum-free medium with transfection reagent according to the manufacturer’s instructions. 200 nM siRNA were used for transfection in each well. DH were added in well after transfected for 48 h. The cells were finally collected for further Western blot and morphological assessment. The sequences of the ribonucleotides used for GSDMD silence were as follows (sequence 5’→3’): 1. GCCGCAUGUGUGCACUCUA and UAGAGUGCACACAUGCGGC; 2. GCAGGAGCUUCCACUUCUA and UAGAAGUGGAAGCUCCUGC (TSINGKE Biological Tech. China).

### Western blot analysis

Cells lysates were prepared as previously described [22]. Cells were lysed with RIPA buffer (pH 7.4 Tris buffer, 150 mM NaCl, 1% NP-40, 0.5% sodium deoxycholate, 0.1% SDS, protease inhibitors and phosphatase inhibitors) for 30 min and denatured in sample loading buffer for 5 min at 97 °C. The lysates were separated by SDS-PAGE and transferred onto PVDF membranes (0.22 μm). After being blocked, the membranes were incubated with primary antibodies overnight. Then, the second antibodies (HRP-conjugated secondary antibodies) were incubated and visualized with ECL. The imaging data were quantified using Imaging LabTM software (Bio-Rad). The expression of HMGB1, NLRP3, ASC, and STING protein in the results were all from cell lysates proteins.

### Real-time Quantitative PCR (qPCR)

The total RNA was extracted from cultured cells by Trizol reagent (Invitrogen), then reversely converted into complementary DNA (cDNA) using cDNA Synthesis Kit (MBI Fermentas, Inc). qPCR was performed using SYBR premix Ex Taq II Kit (Takara Biotechnology) in a Bio-Rad CFX96 system. The primer sequences were listed in Supplementary Table 1, and 18S was used as a loading control for corresponding data.

### Lactate dehydrogenase (LDH) release assay

The LDH release from PANC-1 cells after different treatment were determined using LDH cytotoxicity assay kit, according to the manufacturer’s protocol. Briefly, the supernatant of culture cells was collected in a 96-well plate and the reaction mixture was added in. The OD value was measured at 490 nm using plate reader.

### Measurement of intracellular ROS generation

The peroxide-sensitive fluorescent probe DCFH-DA was utilized for intracellular ROS determination. The DH treated cells were incubated with 5 μM of DCFH-DA for 30 min at 37 °C. After washing twice with PBS, the cells were suspended in PBS and analyzed with fluorescence microscopy (Leica Microsystems, United States). The positive staining index was determined by calculating the positive staining area normalized to the total field area via Image J.

### Detection of Mitochondrial ROS

PANC-1 cells were treated with vehicle or DH for 12 h. Then the medium was removed and the cells were incubated with 1 μM MitoSox for 20 min at 37 °C. The positive fluorescence of the probes was recorded by fluorescence microscopy. The positive staining index was determined by calculating the positive staining area normalized to the total field area via Image J.

### Time-lapse imaging microscopy

Live-imaging was performed using Leica SP8 (Leica) imaging microscopes. The images were captured within an environmental chamber set to maintain a temperature of 37 °C and supplied with a humidified stream of 5% CO_2_ air. PANC-1 cells were seeded in 12-well plates and pretreated with MitoSOX. Imaging commenced immediately after the addition of DH to the cells. Images were captured at 2 min intervals over 12 h.

### Immunofluorescence staining (IF)

PANC-1 cells were cultured on confocal dishes and fixed in 4% paraformaldehyde for 15 min after being treated with vehicle or DH for the indicated time points. 0.1% triton X-100 was added in for permeabilization (15 min) after washing cells with PBS for 3 times. Then cells were blocked with 1% BSA for 30 min and incubated with primary antibody (Tom20, dsDNA, HMGB1) overnight in 4 °C. Finally, cells were incubated with the secondary antibody (secondary goat anti-rabbit Alexa Fluor 555) for 1 h in the dark at RT. The images were captured by the confocal fluorescent imaging system (Zeiss 880, Germany) after the cells were counter stained with DAPI and mounted with mounting medium.

### Quantification of extracellular DNA

Extracellular DNA was detected as previously described [23]. The PANC-1 cells were treated with DH or vehicle for 12 h, and the nuclear dye (TB Green) was added in to stain extracellular DNA. The final concentration of TB Green was 5 μM. We obtained quantitative measurements of extracellular DNA by analyzing the fluorescence intensity (ex: 500/em:530) of the culture using plate reader.

### cGAS CHIP

This method was conducted as previously described with minor modification [24]. 1 × 10^^7^ PANC1 cells were stimulated with DH or not for 6 h. Cells were fixed with a final concentration of 1% formaldehyde for 10 min. Glycine (0.125 M) were added to stop the fixation. Cells were washed by cold PBS for 2 times and were lysed in RIPA buffer for 15 min. The cell lysates were sonicated with a bioruptor pico using 20 cycles 30 s on 30 s off. Anti-cGAS antibody was added in to precipitate the bound DNA overnight in 4 °C. Protein A/G magnetic beads were added into the lysates and incubated for another 8 h in 4 °C. Samples were decrosslinked by adding 500 mM NaCl and addition of proteinase K overnight at 65 °C. QPCR was used to analyze the mitochondria DNA.

### Data collection and preprocessing

We retrieved transcriptome data of GSDM family genes in pancreatic cancer patients from the TCGA-GDC data portal. Utilizing the package “DESeq2 (version: 1.42.1)” in R environment (version:4.3.3), we analyzed these datasets to examine the mRNA expression profiles.

### Histological examination

The liver tissue samples were fixed in 10% paraformaldehyde, eluted by graded alcohol series, embedded in paraffin and 5-micron sections were stained for HE staining. After suffered dewaxing and dehydration successively, the slices were stained with hematoxylin-eosin and were mounted by neutral balata after relived the process of dehydration and dewaxing. Images were obtained using an Slide Scan System(SQS-40P, SHENGQIANG TECHNOLOGY, scanner at x20 magnification).

### Statistical analyses

All statistical analysis was performed using GraphPad Prism 9 software. Randomization was done for the animal grouping. There are no exclusion criteria, except absence of dead mice data. *N* indicates biological replicates throughout the study, and N. Details of number of replicates are provided in the individual figure legends. The values were expressed as mean ± SEM or SD and *P* < 0.05 were considered significant. To analyze the differences between two groups, *unpaired t*-test was used. For data normalized with control group (control is 1), one sample *t*-test against hypothetical value 1 was used to compare. All statistical tests were two-sided. For multiple group comparison, multiple *t*-test followed by Bonferroni correction or one-way analysis of variance was used. Sample size were determined based on previous experience with these types of experiments [25–27]. No statistical methods were applied to predetermine the sample size for in vivo study. The variance was similar between the groups that are being statistically compared.

## Supplementary information

Supplemental Material

Supplementary video 1

## Data Availability

The datasets used and/or analyzed during the current study are available from the corresponding author upon reasonable request.
