# Supplementary material for: Dronedarone hydrochloride (DH) induces pancreatic cancer cell death by triggering mtDNA-mediated pyroptosis
Source: Cell Death Dis. 2024 Oct 2;15(10):725. doi: 10.1038/s41419-024-07102-w (PMC11447222; doi:10.1038/s41419-024-07102-w)

Supplementary data

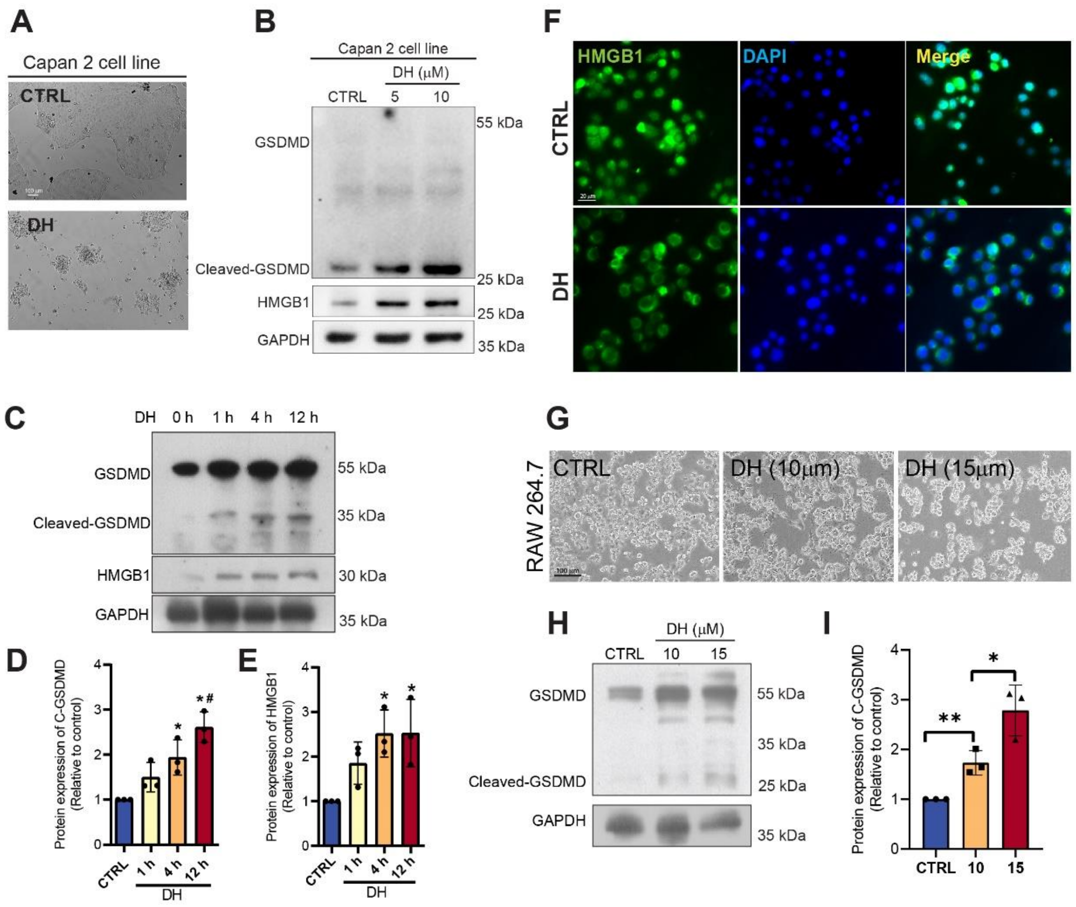

Supplementary Figure 1. A. Morphology observation of Capan 2 cell lines after treatment of vehicle or DH (10 μM). **B.** Representative Western Blot images of pyroptosis-related proteins expression in Capan 2 cells after DH treatment. **C.** Representative Western Blot images of pyroptosis-related proteins expression in PANC 1 cells after DH treatment for different time points. **D-E.** Quantification of cleaved-GSDMD, HMGB1 protein expressions in **C**. *n* = 3. **F.** Representative fluorescent images of PANC1 cells with or without DH treatment (1 h). Scale bar, 20 μm. Green, HMGB1; Blue, DAPI. **G.** Representative images of RAW 264.7 cells with or without DH treatment. Scale bar, 100 μm. **H.** Representative Western Blot images of pyroptosis-related proteins in PANC1 cells after DH treatment or not. **I.** Quantification of cleaved-GSDMD protein expression in **G**. Data was shown as mean ± SD. Unpaired t-test was used. *p < 0.05; ** p < 0.01.

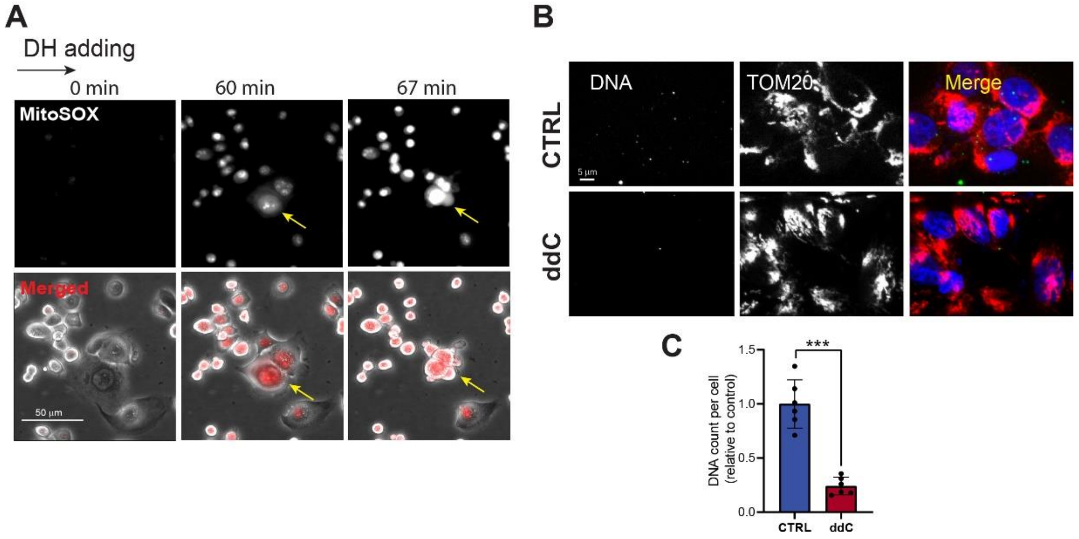

Supplementary Figure 2. A. Representative time-lapse images of PANC1 cells after treating with DH. Cells were pretreated with MitoSox. Scale bar, 50 μm. Red, MitoSox. **B.** Representative immunofluorescent images of PANC1 cells with or without ddC treatment. Scale bar, 5 μm. **C.** Quantification of DNA positive staining area in images in B. *n*=6. Data was shown as mean ± SD. Unpaired t-test was used. *** $p < 0.001$.

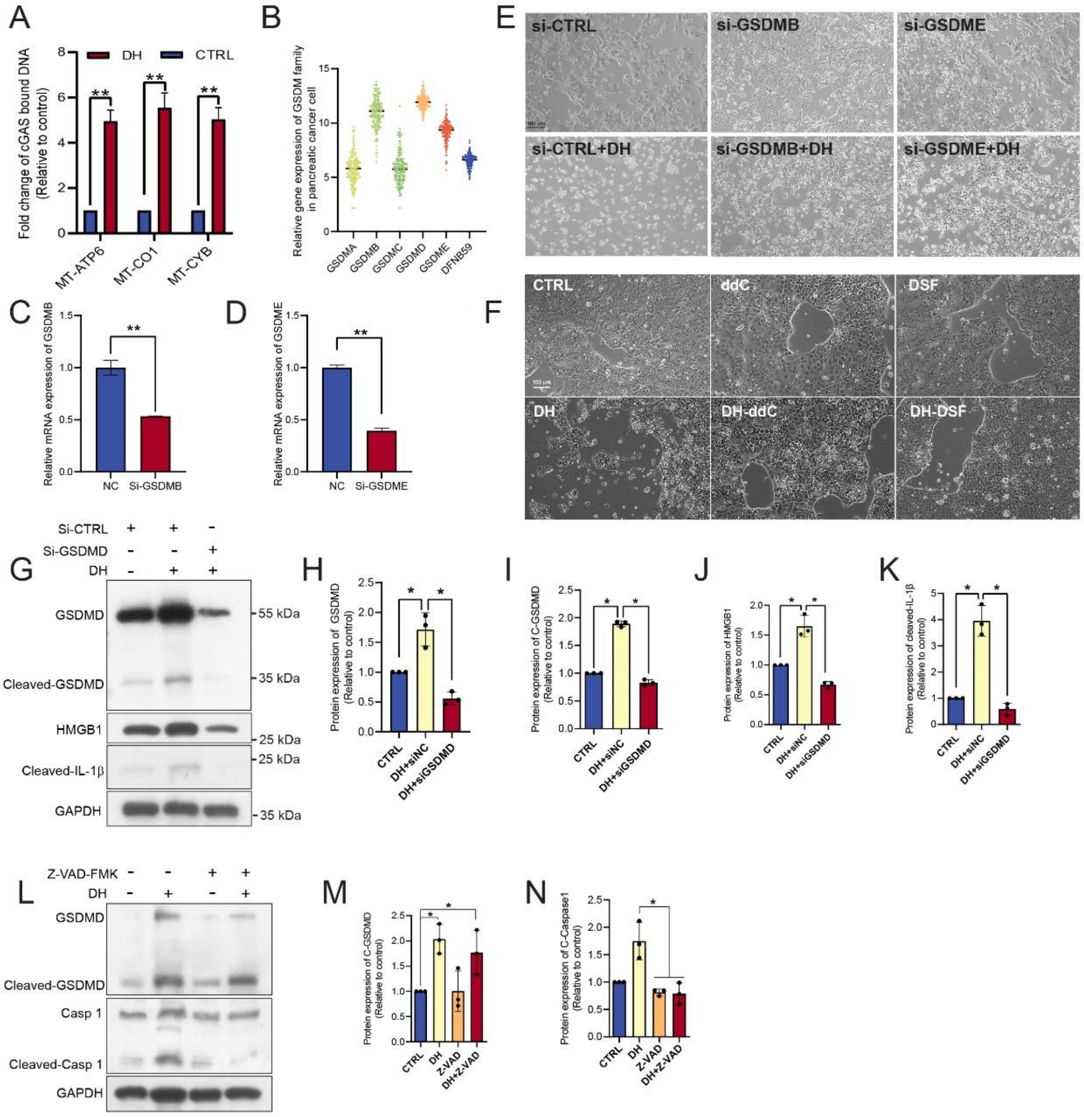

Supplementary Figure 3. A. Comparison of cGAS-bound mtDNA after DH treatment. *MT-ATP6*, *MT-CO1*, and *MT-CYB* are all mitochondrial genes. **B.** Comparison of gene expressions of GSDM family in pancreatic cancer in TCGA database. **C.** Comparison of gene expressions of GSDMB after transfecting GSDMB siRNA. *n*=3. **D.** Comparison of gene expressions of GSDME after transfecting GSDME siRNA. *n*=3. **E.** Representative images of cell morphology after silencing different GSDM genes and treatment of DH or not. **F.** Representative images of cell morphology after different treatment. **G.** Representative Western Blot images of pyroptosis-related proteins in PANC1 cells after silence GSDMD (oligo 2). **H-K.** Quantification of GSDMD, cleaved-GSDMD, HMGB1, and cleaved-IL-1β protein expressions in **G**. *n* = 3. **L.** Representative Western Blot images of pyroptosis-related proteins in PANC1 cells after different treatment. **M** and **N.** Quantification of cleaved-GSDMD and Caspase 1 protein expressions in **L**. Data was shown as mean ± SD. Unpaired t-test was used. ** p < 0.01.

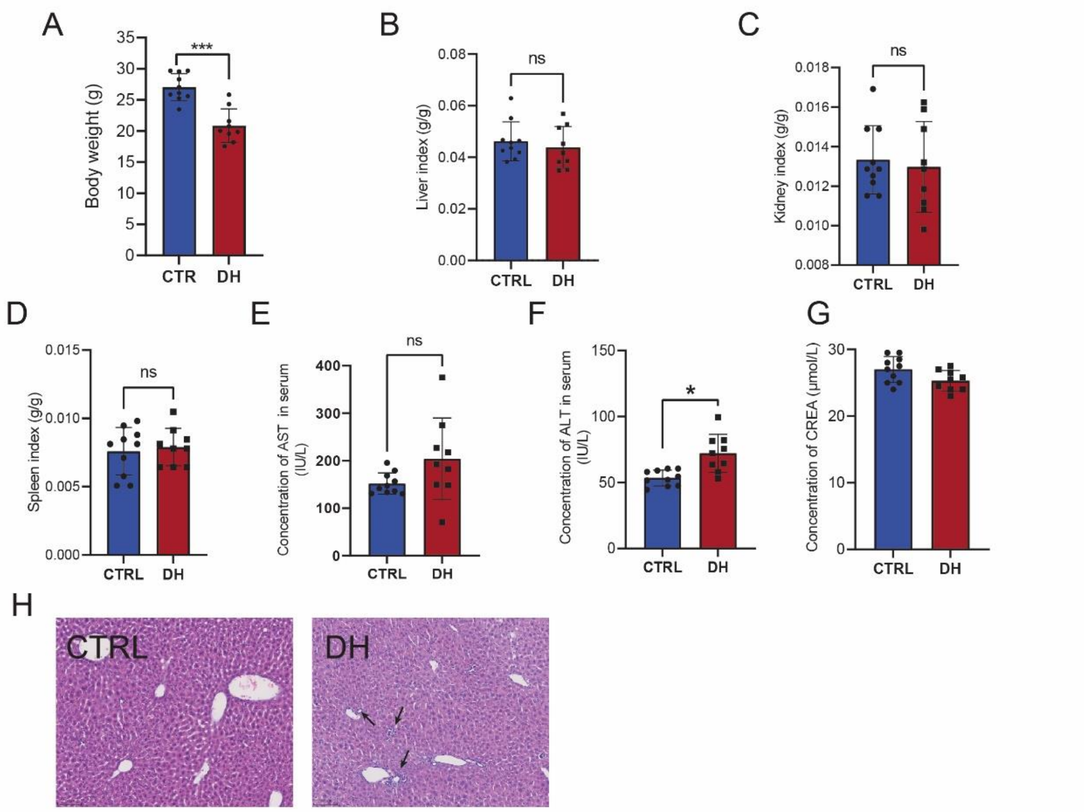

Supplementary Figure 4. Organ weights and determination of liver or kidney function after DH treatment. A. Body weight of nude mice after xenograft PANC-1 cells. Mice were administrated with DH or not. *n*=9. **B-D.** Organ indexes in nude mice with xenograft of PANC-1 cells after administration of DH or not. B, liver index (*n*=9); C, kidney index (*n*=9,10); D, spleen index (*n*=9,10). **E-F.** Determination of AST or ALT concentrations in serum of nude mice with xenograft of PANC-1 cells after administration of DH or not. E, AST (*n*=9); F, ALT (*n*=9,10) **G.** Determination of CREA concentrations in serum of nude mice with xenograft of PANC-1 cells after administration of DH or not. (*n*=9,10). **H.** HE staining of liver section from mice with or without DH treatment. Infiltration sites of Inflammatory cells (black arrow). Data was shown as mean ± SD. Unpaired *t*-test was used. ** $p < 0.01$; *** $p < 0.001$.

Supplementary Table 1. The sequences of primers (SYBR) for RT-qPCR

Gene	Sequences(5'-3')	
18S	F: 5'-TCTTCATTGACCAAGGAAATCGG-3'	R: 5'- TCCGGGGTGCATTATCTCTAC-3'
MT-ATP6	F: 5'- ACCACAAGGCACACCTACAC-3'	R: 5'- TATTGCTAGGGTGGCGCTTC-3'
MT-CO1	F: 5'- ATACCAAACGCCCCTCTTCG-3'	R: 5'- TGTTGAGGTTGCGGTCTGTT-3'
MT-CYB	F: 5'- AGCCAACCCCTTAAACACCC-3'	R: 5'- TGTTAGGGACGGATCGGAGA-3'
GSDMB	F: 5'- GAAACTCTGGAGACGGTAAAGG-3'	R: 5'- GATAGCTCAGGACCCGATTTG-3'
GSDME	F: 5'- AACTCCAGATCATTCCCACAC-3'	R: 5'- CTGCACAATCCCAAACCTTTC-3'

Fig2.C

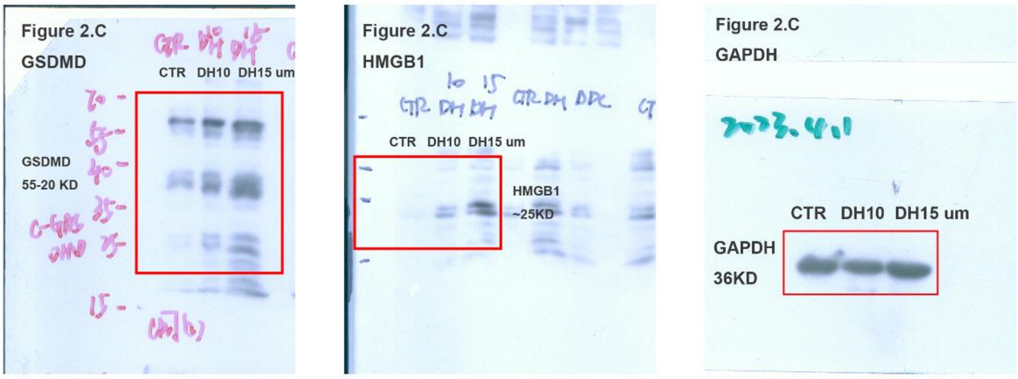
Figure 2.C
GSDMD
CTR DH10 DH15 um
GSDMD
55-20 KD
Figure 2.C
HMGB1
CTR DH10 DH15 um
HMGB1
~25KD
Figure 2.C
GAPDH
CTR DH10 DH15 um
GAPDH
36KD

Fig2.F

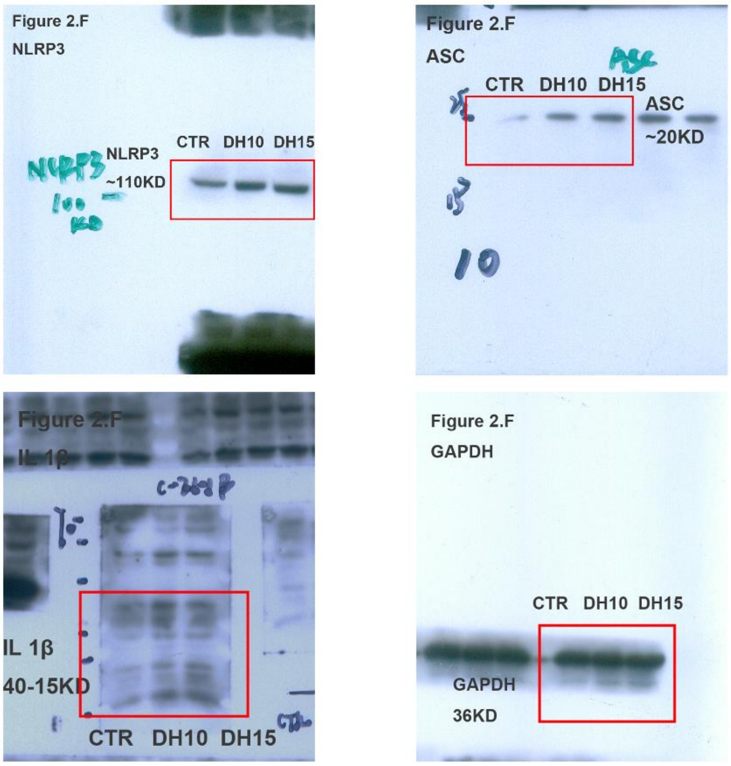
Figure 2.F
NLRP3
CTR DH10 DH15
NLRP3
~110KD
Figure 2.F
ASC
CTR DH10 DH15
ASC
~20KD
IL 1β
40-15KD
CTR DH10 DH15
Figure 2.F
GAPDH
CTR DH10 DH15
GAPDH
36KD

Fig3.A

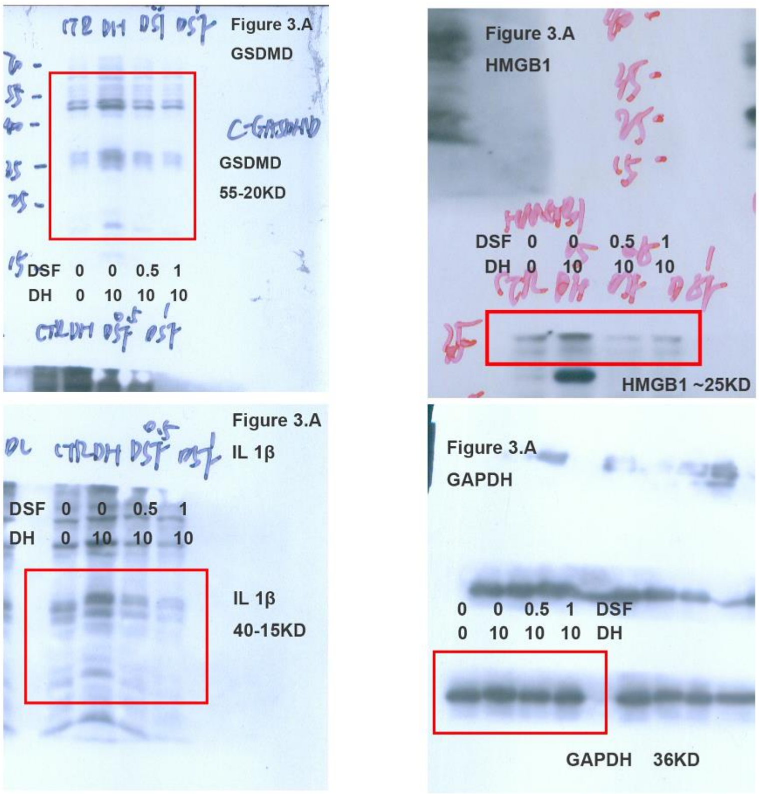
Figure 3.A
GSDMD
GSDMD
55-20KD
DSF 0 0 0.5 1
DH 0 10 10 10
Figure 3.A
HMGB1
DSF 0 0 0.5 1
DH 0 10 10 10
HMGB1 ~25KD
Figure 3.A
IL 1β
DSF 0 0 0.5 1
DH 0 10 10 10
IL 1β
40-15KD
Figure 3.A
GAPDH
0 0 0.5 1 DSF
0 10 10 10 DH
GAPDH 36KD

Fig3.F

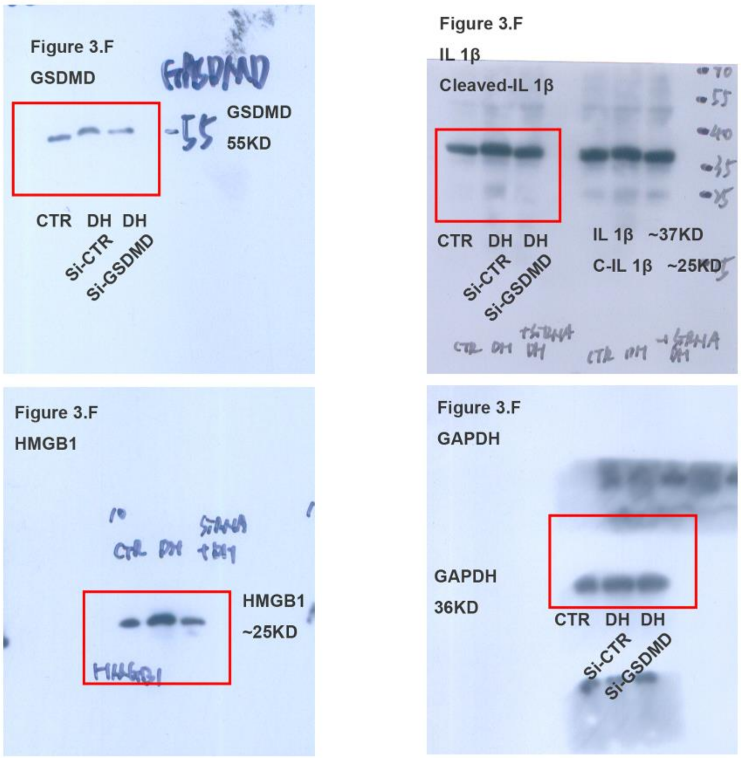
Figure 3.F
GSDMD
GSDMD
55KD
CTR DH DH
Si-CTR
Si-GSDMD
Figure 3.F
IL 1β
Cleaved-IL 1β
CTR DH DH
Si-CTR
Si-GSDMD
IL 1β ~37KD
C-IL 1β ~25KD
Figure 3.F
HMGB1
HMGB1
~25KD
Figure 3.F
GAPDH
GAPDH
36KD
CTR DH DH
Si-CTR
Si-GSDMD

Fig5.C

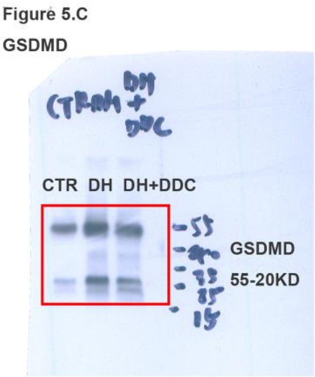
Figure 5.C
GSDMD
CTR DH DH+DDC
GSDMD
55-20KD

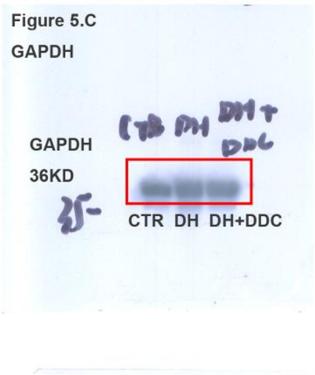
Figure 5.C
GAPDH
GAPDH
36KD
CTR DH DH+DDC

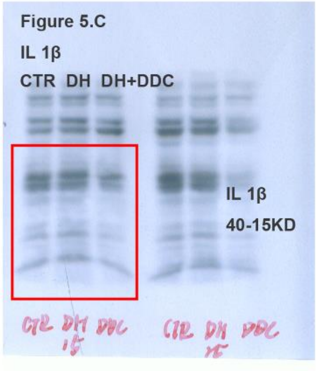
Figure 5.C
IL 1β
CTR DH DH+DDC
IL 1β
40-15KD

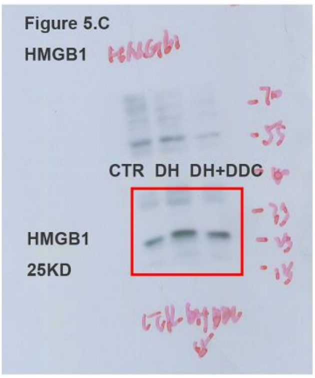
Figure 5.C
HMGB1
CTR DH DH+DDC
HMGB1
25KD

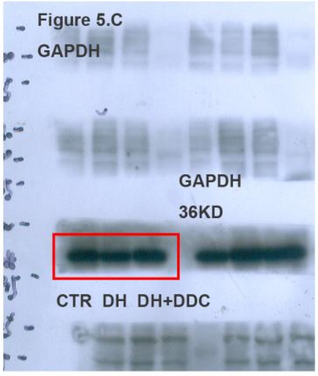
Figure 5.C
GAPDH
GAPDH
36KD
CTR DH DH+DDC

Fig6.C

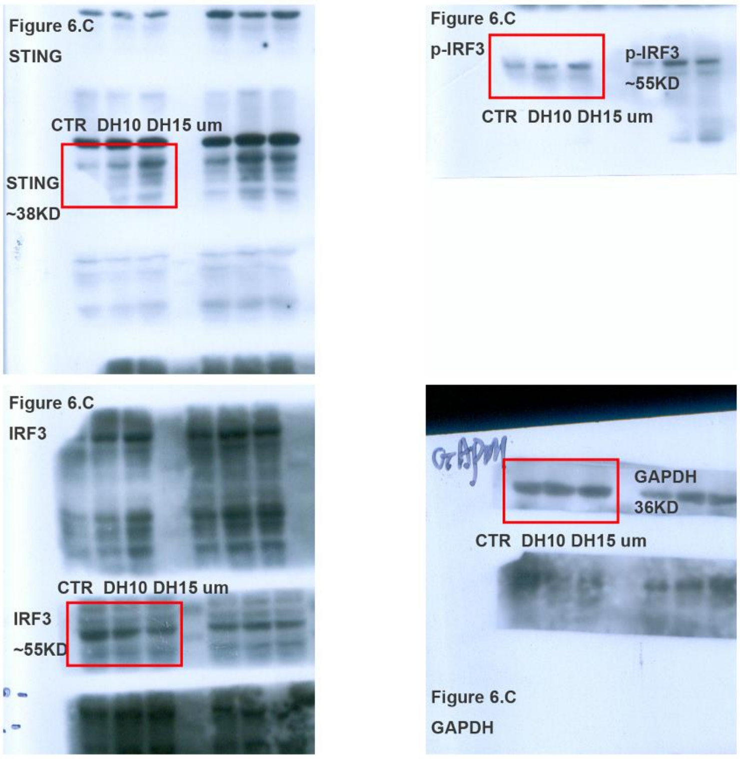
Figure 6.C
STING
CTR DH10 DH15 um
STING
~38KD
Figure 6.C
p-IRF3
p-IRF3
~55KD
CTR DH10 DH15 um
Figure 6.C
IRF3
CTR DH10 DH15 um
IRF3
~55KD
GAPDH
36KD
CTR DH10 DH15 um
Figure 6.C
GAPDH

Fig6.G

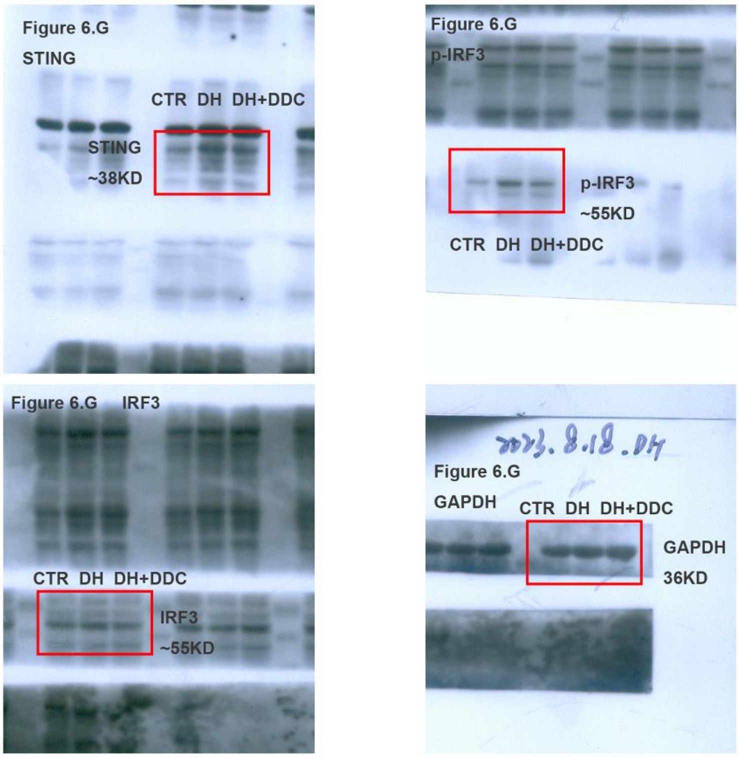
Figure 6.G
STING
CTR DH DH+DDC
STING
~38KD
Figure 6.G
p-IRF3
p-IRF3
~55KD
CTR DH DH+DDC
Figure 6.G IRF3
CTR DH DH+DDC
IRF3
~55KD
Figure 6.G
GAPDH
CTR DH DH+DDC
GAPDH
36KD

Fig7.D

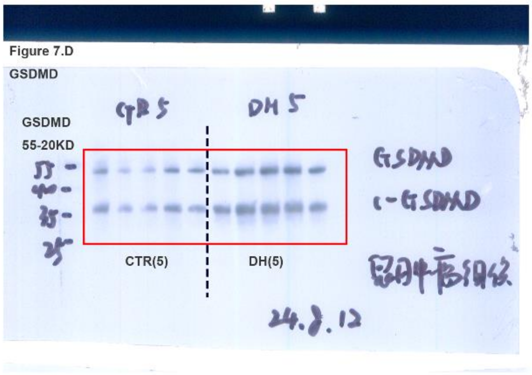

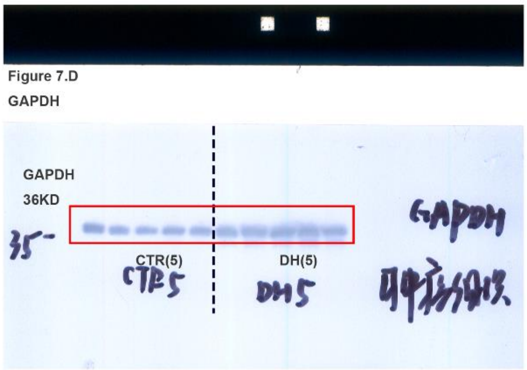

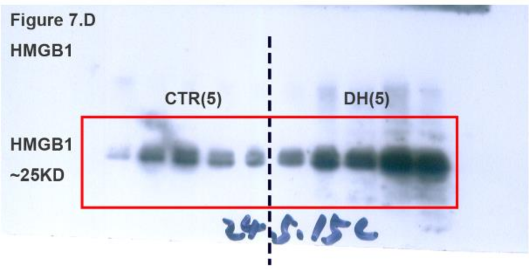

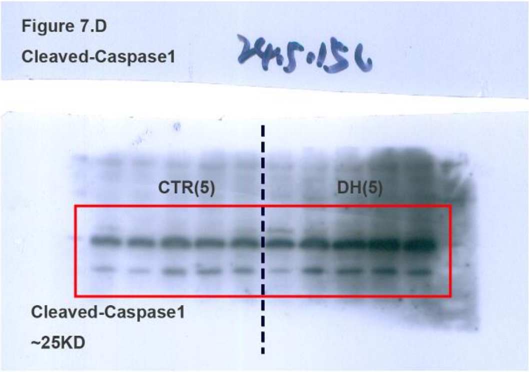

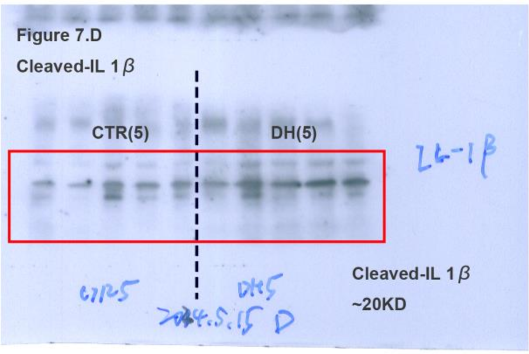

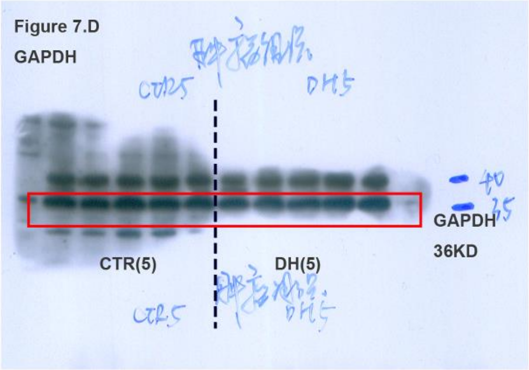

SFig1.B

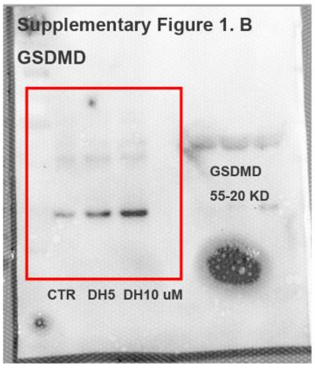

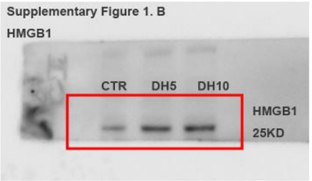

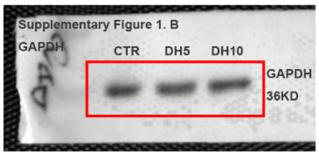

SFig1.C

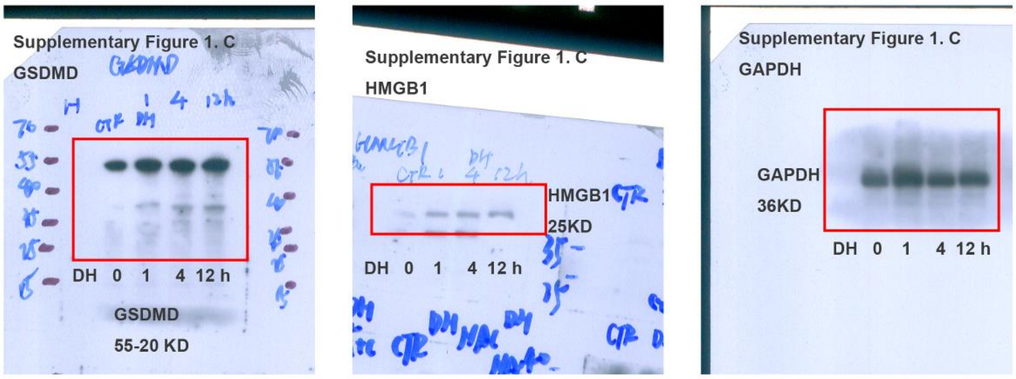

SFig1.H

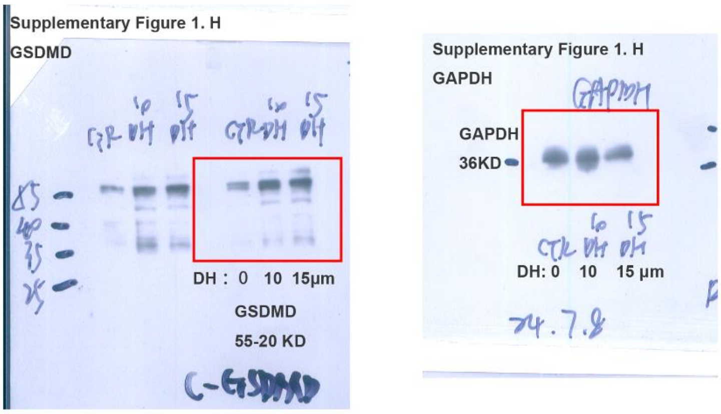

SFig3.G

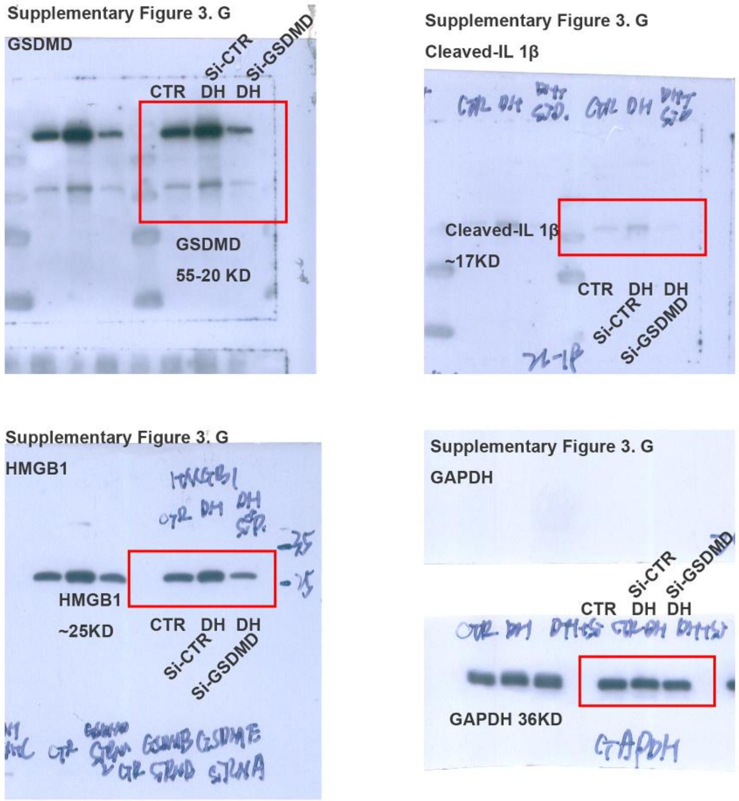

SFig3.L

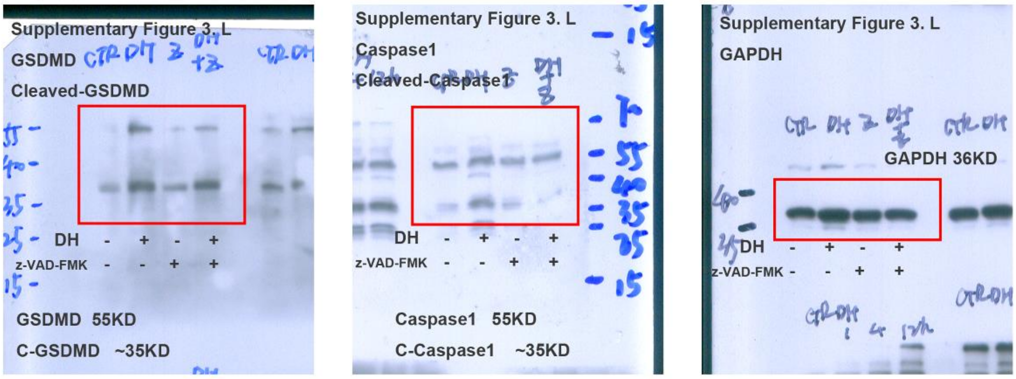

Supplement: Supplementary file 1 — Supplemental Material [file 41419_2024_7102_MOESM1_ESM.pdf]
